# Cardiac Tamponade Caused by *Cutibacterium acnes*: An Updated and Comprehensive Review of the Literature

**DOI:** 10.1155/2020/9598210

**Published:** 2020-07-14

**Authors:** Ghina Fakhri, Christelle Tayeh, Ghassan Dbaibo, Omar El Sedawy, Nour Abdul Halim, Fadi Bitar, Mariam Arabi

**Affiliations:** ^1^Division of Cardiology, Department of Pediatrics and Adolescent Medicine, American University of Beirut Medical Center, Beirut, Lebanon; ^2^Division of Infectious Diseases, Department of Pediatrics and Adolescent Medicine, American University of Beirut Medical Center, Beirut, Lebanon

## Abstract

Bacterial pericarditis is a critical diagnosis caused by a wide range of organisms including *Streptococcus pneumoniae* and other anaerobic organisms like *Cutibacterium acnes* which has been gaining more importance as a causative organism. Cutibacterium species are Gram-positive microaerophilic rods that constitute part of the normal flora of skin and mucosal membranes. The incidence of pericarditis caused by this organism is underreported as it is often dismissed as a skin flora contaminant. However, if left untreated, *Cutibacterium acnes* can cause pericarditis with serious complications. In this paper, we present a comprehensive review of the literature regarding pericarditis caused by *Cutibacterium acnes* along with a case presentation from our institution. In our institution, a 20-year-old man with history of atrial septal defect presented with chest pain radiating to the back along with symptoms of upper respiratory tract infection including headaches and myalgia. Electrocardiogram was remarkable for diffuse low-voltage waves. Echocardiography revealed a large pericardial effusion with tamponade features. Pericardiocentesis drained 1.2 L of milky fluid. Pericardial fluid analysis grew *Cutibacterium acnes* after being cultured for 8 days. The patient received 3 weeks of IV penicillin followed by 3 weeks of oral amoxicillin along with nonsteroidal anti-inflammatory agents and colchicine with no recurrence. Pericarditis caused by *Cutibacterium acnes* requires a high clinical suspicion since isolation of this organism can be dismissed as a skin flora contaminant. Literature review reveals that this infection may be underdiagnosed and underreported. Prompt diagnosis may lead to timely initiation of antibiotics which can help prevent devastating complications like constrictive pericarditis. Prospective studies are needed to evaluate the true incidence and prevalence of this disease.

## 1. Introduction

Infectious pericarditis remains a very critical diagnosis, and many infectious organisms can be the culprits. Bacterial pericarditis is a subset of infectious pericarditis that is an important cause of morbidity and mortality as it progresses rapidly and is fatal if left untreated [1, 2]. In the current era of modern antibacterial therapy, the incidence of bacterial pericarditis decreased to 1 in 18,000 individuals [3–5]. Moreover, there has been a change in the spectrum of pathogens responsible for bacterial pericarditis. Around the time of World War II, the pathogens consisted of Gram-positive organisms and has transitioned to include Gram-negative as well as anaerobic ones. Keeping a high index of suspicion is crucial to avoid diagnostic delay and initiate proper treatment, as there are numerous critical complications such as constrictive pericarditis, tamponade, and left ventricular pseudoaneurysm [3, 4, 6]. Early detection is crucial to allow timely initiation of the appropriate antibacterial treatment, which, if administered early, can prevent these complications.

The usual organism responsible for pericarditis in the preantibiotic era was *Streptococcus pneumoniae* and was soon followed by facultative aerobic Gram-negative bacilli including *Escherichia coli* and *Klebsiella pneumoniae*. Recently, the spectrum has shifted to include anaerobic bacteria such as *Prevotella*, *Bacteroides fragilis*, and *Peptostreptococcus* species and other rare organisms like *Cutibacterium acnes* (C. acnes) [6]. *C. acnes* is considered to have a low virulence but has been recognized recently as a cause of serious infections like endocarditis and pericarditis [7, 8]. The incidence of pericarditis caused by *C. acnes* has been increasing worldwide with scarce literature reported on the matter. *C. acnes* is a slowly growing Gram-positive microaerophilic rod that requires no less than 7 days to grow in culture. In this manuscript, we report *Cutibacterium acnes* infection in an adult with pericarditis and present an updated comprehensive review of *C. acnes* causing pericarditis.

## 2. Methodology

This review was finalized in December 2019. The literature review is up to date and articles were critically appraised for validity and relevance. A comprehensive search was conducted in PubMed, Medline (1946–2018), and Google Scholar for the presence of grey literature. Articles were included if they were published in the English language and reported on the incidence or prevalence of *Cutibacterium acnes* in patients with pericardial effusion or pericarditis. No limitations were made on year of publication, age, or country of origin. The search strategy consisted of two concepts. The first concept regarding *Cutibacterium acnes* was searched using MeSH terms and keywords for the following: *Propionibacterium acnes, Cutibacterium acnes, Propionibacterium, cutibacterium*, C Acnes, and P acnes. As for the second concept, it was searched using the following MeSH terms and keywords: pericardial effusion, effusion, pericarditis, and tamponade. A total of 6 articles reported on 10 *Cutibacterium acnes* cases and their association with pericarditis. A table summarizing their characteristics is available (Table 1).

## 3. Case Presentation

**Figure 1:**
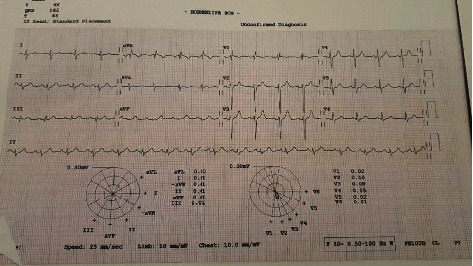
Electrocardiogram showing low voltage among the majority of the leads.

**Figure 2:**
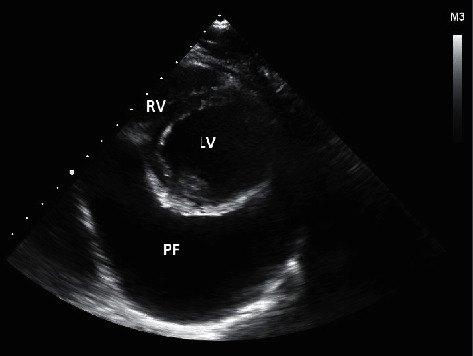
Echocardiography, short-axis view of the heart showing the LV and large pericardial effusion. LV: left ventricle; PF: pericardial fluid; RV: right ventricle.

**Figure 3:**
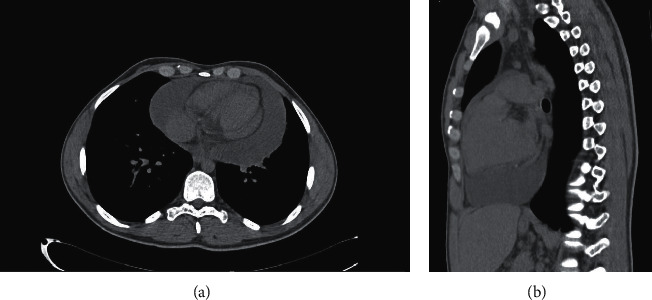
Axial (a) and coronal (b) sections of a nonenhanced CT chest showing pericardial effusion.

**Figure 4:**
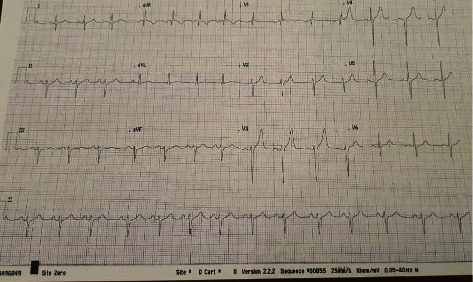
Electrocardiogram after treatment for pericardial effusion revealing normalization of the voltage in the majority of the leads.

A 20-year-old man, with a history of pulmonary stenosis and atrial septal defect (ASD) status post-ASD closure, presented with respiratory tract infection symptoms, myalgia, chest pain radiating to the back, and headache of two weeks duration. In addition, he reported nasal congestion, dyspnea, and a nonproductive cough. He did not have rhinorrhea, sneezing, fever, or any other sign or symptom. Physical exam and vital signs were remarkable for few abnormalities. Temperature taken from the axilla was 36.9°C, oxygen saturation was 94%, pulse was 97 beats per minute, respiratory rate per minute was 18, and blood pressure was 143/84 mmHg. The physical exam was pertinent for distant heart sounds and a grade II/VI systolic ejection murmur without friction rub. In addition, he was noted to have few comedones on his upper back that were not inflamed or infected in nature and have been present for a long time and left untreated. Electrocardiogram (EKG) revealed low voltage waves (Figure 1). Transthoracic electrocardiogram revealed mild-to-moderate pulmonary stenosis and a large pericardial effusion with right ventricular collapse suggestive of tamponade (Figure 2). Chest X-ray revealed increased cardiac silhouette, and this was followed up with a CT of the chest without contrast which revealed a moderate-to-large pericardial effusion with maximal thickness up to 3.2 cm inferiorly and established the diagnosis of pericarditis (Figure 3). Complete blood count revealed leukocytosis (WBC count: 15,100/cu.mm), with 67% neutrophilia. Blood culture, on the other hand, revealed no growth. A full PCR respiratory panel performed upon presentation, of 22 pathogenic agents (nucleic acid from Adenovirus, Coronavirus, Rhinovirus, Enterovirus, Human Metapneumovirus, Middle East Respiratory Syndrome Coronavirus, Parainfluenza virus, Respiratory Syncytial virus, *Bordetella pertussis*, *Chlamydia pneumoniae,* and *Mycoplasma pneumoniae)* was negative as well. Initially, erythrocyte sedimentation rate was 8 mm/hour and C-reactive protein was 5.3 mg/L. Empirically, the patient was started on Ibuprofen 400 mg three times daily. To alleviate the right ventricular collapse, immediate drainage was undertaken. Pericardiocentesis was performed draining 1.2 L of a milky fluid. The fluid analysis revealed the following: WBC cell count 63/cu.mm with many macrophages on microscopy, LDH 348 IU/L, protein 71.7 g/L, glucose 20 mg/dL, and albumin 48.9 g/L. A work-up looking for the rheumatological diseases was done and was negative (ANA, rheumatoid factor, anti-CCP2, anti-centromere antibodies, anti-Scl70 antibodies, anti-smith antibodies, and anti-ds DNA antibodies). Cardiac markers including troponin, creatine phosphokinase, and CK-MB were checked and were negative. Fluid testing for mycobacterium tuberculosis, fungi, and other bacteria was negative. After the drainage, the patient was switched to ketoprofen 100 mg twice daily as well as colchicine 1 mg once daily as part of the standard of care for patients with pericardial effusion. The treatment with colchicine and ketoprofen lasted for a total of 3 months. After 8 days of incubation, the fluid culture revealed moderate growth of *Cutibacterium acnes*. As such, the patient received 3 weeks of parenteral penicillin G. Follow-up echocardiography revealed a residual rim of pericardial effusion. The patient was given 3 more weeks of oral amoxicillin. His last follow-up at the end of the antibiotic course showed complete resolution of his symptoms and disappearance of the residual pericardial effusion. Repeat EKG revealed normalization of the voltage on all the leads (Figure 4). Vital signs returned to baseline. Heart rate was 80, blood pressure was 125/74 mmHg, and oxygen saturation was 100%. The ketoprofen and colchicine were discontinued after complete resolution of the effusion. The repeat CRP on two separate occasions was normal (<2.5 mg/L). He did not require a continuous pericardial drain nor surgical intervention.

## 4. Discussion

### 4.1. Incidence and Etiology

Bacterial pericarditis has become a rare entity in our era where the incidence decreased from 1/254 to 1/18,000 persons, attributed to the widespread use of antibiotics [3–5]. Early on, *Staphylococcus* and *Streptococcus* were very common, followed by *Haemophilus* and *Mycobacterium tuberculosis*. Bacterial pericarditis has recently witnessed an increased incidence of anaerobic infections from *Prevotella* species and *Cutibacterium acnes* reaching 40% of all cases [3, 4]. *Cutibacterium* species are vital members of the normal microbial flora which reside in the pilosebaceous follicles in the human skin, conjunctiva, oral cavity, external ear, and intestinal tract [13, 14]. *C. acnes* is commonly dismissed as a skin flora organism, but it has been associated with serious infections of the skin, soft tissue, cardiovascular system, and other implant-associated infections. *C. acnes* infection of the skin is acquired in the following manner: inflammatory mediators released into the skin trigger an inflammatory state, altered keratinization process, comedone formation, increased androgens, sebum production, and, finally, colonization of the follicle. This pathogen has the ability to trigger an inflammatory cytokine release in macrophages thereby modulating the immune state. The optimal environment for *C. acnes* is provided by the skin's anaerobic and lipidic conditions. There are a number of virulence factors produced by this organism such as the ability to cause bacterial seeding, manipulation of host immune system, and biofilm formation, all of which contribute to disease evolvement. In patients who undergo dental or other surgeries, *C. acnes* can cause seeding and deep bacterial infection [15, 16]. This organism is a Gram-positive, catalase-positive microaerophilic anaerobic-aerotolerant pleomorphic bacillus. It has an ability to form biofilms on many devices and to survive up to 8 months in anaerobic conditions or human tissue with low oxidation status [10, 17]. Recovering *C. acnes* from blood, fluid, or tissue cultures may be difficult as it requires 7 to 14 days for proper isolation. Moreover, the culture time might extend to 3 weeks in both aerobic and anaerobic atmospheres to grow *C. acnes* [17, 18]. *C. acnes* has a solid reputation for being both slow growing and of low virulence, but there have been recent emerging reports on its involvement in periodontitis, pericarditis, endocarditis, as well as infections in the central nervous system, and prosthetic devices [3, 4, 19]. It can be considered a major contaminant, but physicians should not dismiss repeated isolation of *C. acnes*. In addition, microscopic examination of tissue infected with *C. acnes* reveal inflammatory infiltration and fibrosis which further confirms that *C. acnes* has an immunostimulatory effect on the mononuclear phagocyte system [11, 16]. A comprehensive literature review revealed 61 cases of pericarditis caused by *C. acnes* of which only 10 had supportive demographic or clinical information presented in Table 1 [1]. Patients diagnosed with *C. acnes*-induced pericarditis are usually adults with a mean age of 50.6 years. Our patient has been the youngest of all patients diagnosed with pericarditis caused by *C. acnes*.

### 4.2. Risk Factors

Bacterial pericarditis has always been considered as a complication to pneumococcal pneumonia in the old era, but, currently, most cases are associated with compromised immunity, cardiac surgery, and malignancy, among others [20–23]. The pericardium, unlike other organs, is considered a rare primary site of infection and there are four different ways believed to aid in spreading the infection: (1) local extension such as endocarditis, (2) direct extension of an intrathoracic process such as pneumonia, (3) perforating injury to the chest wall, and (4) hematogenous spread [3, 4, 6, 12, 13]. The frequency of an underlying bacteremia depends on several factors such as the host's immune system, skin colonization by *C. acnes*, presence of dental caries, or infections. In addition, there are sites in the human body where *C. acnes* colonization is heavier than others. For example, areas of the under arm and around the shoulder carry a higher infection risk and higher likelihood of bacteremia if the patient goes into surgery than sites of the hip. That is the case because there are more sebaceous glands around the shoulder area than there are around the hip which increases the chance for bacterial seeding and subsequent infection [24]. The frequency of *C. acnes* bacteremia is reported in 10% of periprosthetic joint infection with a higher predilection for shoulder surgeries. The other famous sites of infection include breast implants and cardiac sites especially the valves. In a large study, authors identified 24 out of 1325 (1.8%) patients with infective endocarditis to have had *C. acnes* positive culture. Of those patients, *C. acnes* blood culture was positive in only 12.5% of them [25–27]. In a study of 166 patients whose blood cultures yielded anaerobic bacteria, authors found that none of the 53 patients with *Cutibacterium* bacteremia had clinically significant disease [25]. In another study, the incidence of clinically significant *C. acnes* bacteremia was reported to be 3.5% but none of their patients had pericarditis [24].

In our patient, we do not believe that his infection started as a serious one. Despite the dyspnea, he was able to ambulate and breathe comfortably. He had stable vital signs and was admitted to the regular hospital ward without the need for respiratory support or intensive care. We reached the conclusion that his symptoms were not solely caused by a respiratory infection since he had scarce upper URI symptoms, respiratory panel was negative, and chest X-ray or CT did not reveal a pulmonary infection. As such, we believe that the symptoms were arising from the pericarditis instead of a respiratory infection.

Moreover, certain conditions increase the likelihood of acquiring *C. acnes* such as chest wall trauma, tooth decay and dental surgeries, immunosuppression, cardiac surgery, prosthetic heart valves, ventriculoperitoneal shunts, and other foreign bodies [8, 9, 18, 28–30]. While our patient had comedonal acne, he did not suffer from other skin infections, dental caries, or abscesses.

### 4.3. Clinical Presentation

Commonly, bacterial pericarditis presents with high-grade fever, chills, shortness of breath, and tachycardia. A high index of suspicion is required because the classic signs of pleuritic chest pain, pericardial friction rub, and electrical alternans are commonly absent. Physicians need to have a high index of suspicion as the classic signs and symptoms of pleuritic chest pain, electrical alternans, and pericardial friction rub may be absent, but fever is almost always present. Early hemodynamic compromise such as tamponade may be indicated by hypotension [6, 20]. Unfortunately, some patients may present with fever and hypotension only and such a presentation can be misdiagnosed as a septic shock and delay the diagnosis and treatment.

### 4.4. Diagnosis

General laboratory tests may be nonspecific in patients with bacterial pericarditis but anemia and leukocytosis with a left shift are commonly present in addition to increased ESR and CRP [6, 20]. The pauci-inflammatory nature of *C. acnes* may have contributed to the lower-than-expected rise in inflammatory markers in our patient. Chest X-ray might reveal cardiomegaly, pleural effusion, pulmonary infiltrates, or mediastinal widening. On EKG, diffuse ST elevation and PR depression may only occasionally be present [2–6, 31]. Although TTE cannot distinguish between inflammatory effusions and purulent fluid collections, it can facilitate the detection and quantification of fluid. The mere presence of pus draining from a pericardial tube represents 100% sensitivity to bacterial purulent pericarditis. After immediate pericardiocentesis, the fluid should be sent for extensive testing including cell count with differential, gram stain, acid-fast bacillus stain, fungal stain, and culture for both aerobic and anaerobic bacteria. Moreover, performing biochemical analysis can help distinguish between transudates and exudates. Fluid from bacterial pericarditis will reveal a high white blood cell count with predominance of polymorphonuclear cells, low glucose, high protein, and lactate dehydrogenase levels [4, 20, 32, 33]. In our patient, the pericardial fluid that was yielded with the pericardiocentesis was milky or pussy, the sugar was relatively low, and the proteins were elevated. We know, however, that the pericardial fluid that is usually associated with the viral illnesses or rheumatological diseases is usually serous or serosanguinous. The blood WBC count was elevated with a left shift. These findings are most likely suggestive of a bacterial etiology of the pericardial effusion. The WBC was relatively lower than expected in bacterial pericarditis but this is not surprising since this bacterium is pauci-inflammatory in nature. Pericardioscopy and pericardial biopsy can be performed to increase the diagnostic accuracy [6]. As for diagnosing *C. acnes* specifically, it has various characteristics that make the diagnosis challenging. It is a common skin organism and it is frequently dismissed by physicians as a skin flora contaminant. It may also be missed when fluid culture is not incubated for a long time. And as such, it is both underdiagnosed and underreported [13, 34]. After consultation with our infectious diseases department regarding the management of our patient, we believe that his infection with *C. acnes* and its causal relationship to the development of pericardial effusion was genuine. The fluid cytology was indicative of a bacterial infection, fluid culture revealed *C. acnes* growth after 8 days of incubation, other testing for fungi and *tuberculosis* was negative, and the residual rim of effusion fully responded to antibiotics and disappeared. For future patients, it would have been helpful to culture the skin from areas such as the back, neck, underarms, and shoulder for growth and/or colonization of this organism. We did not perform this on our patient, but it might have added insight into the origin of *P. acnes* and its route to cause infectious pericarditis. We believe that this organism was the most likely cause of pericarditis in our patient. We acknowledge that it may be difficult to agree with this diagnosis as it is extremely rare. He received three weeks of IV antibiotics and follow-up revealed a residual rim of effusion, so he was given 3 weeks of oral antibiotics. The total 6-week duration of antibiotics points to the fact that there truly is a bacterial infection causing the pericarditis, one that responds to penicillin. The culture failed to grow any other organism, so we believe that *Cutibacterium acnes* is the most likely cause for the pericarditis.

### 4.5. Prognosis

The mortality of purulent bacterial pericarditis reaches 100% without timely and effective antimicrobial therapy. Even in patients who receive treatment, the mortality approaches 40%. The serious complications that are associated with it are subsequent constrictive pericarditis and cardiac tamponade both of which can cause hemodynamic compromise. Other reported complications consist of aortic mycotic aneurysm, left ventricular pseudoaneurysm, and submitral pseudoaneurysm [3–5, 35–37]. Features that constitute poor prognosis include a delay in diagnosis, failure to initiate treatment, and an immunocompromised status. Proper investigation and timely administration of antibiotics have been shown to prevent several complications [1, 3].

### 4.6. Medical Management

In patients who present with hemodynamic compromise, prompt drainage and placement of a catheter are critical. Antibacterial therapy must be initiated immediately after diagnosis followed by sensitivity-specific medications. Intravenous antibiotics can achieve high concentrations in the pericardial effusion with a peak reaching approximately 2 hours after infusion [38]. The majority of *Cutibacterium* species are susceptible to carbapenems, penicillin, cephalosporins, vancomycin, and aminoglycosides. Expert opinion recommends initiating penicillin G until the treatment can be further guided by antimicrobial susceptibility. Alternatively, combining an antistaphylococcal and an aminoglycoside can be started followed by a tailored therapy. If the infection is critical and life-threatening, a three-drug combination of vancomycin, third-generation cephalosporin, and fluoroquinolone can be given [39]. Metronidazole is the only agent to which *C. acnes* is consistently resistant and should be avoided. Other antibiotics with a relatively higher levels of resistance are clindamycin, erythromycin, and tetracycline [40]. There is no consensus on the required duration of antimicrobial treatment for *C. acnes* but is expected to be prolonged as this organism is intracellular and resists phagocytosis. A minimum of 4 weeks is recommended and should be extended to months in patients who recur [1, 2, 4, 12].

The pericardial space is bordered by the visceral and the fibrous layers. Regardless of the causative agent, whether infectious or postoperatively, inflammation of these layers increases fluid exudate. Inflammation also decreases the normal process of pericardial drainage by the lymphatic ducts. As such, inflammation alone can cause pericardial effusion and the use of nonsteroidal anti-inflammatory drugs (NSAIDs) and colchicine has been the cornerstone in the treatment of patients with acute or recurrent viral or idiopathic pericarditis. That being the case, we cannot exclude the presence of mere inflammation in the pericardial tissue in patients with pyogenic pericarditis [41, 42]. While NSAIDs have been historically used in idiopathic and viral pericarditis, we have shown that their use, in addition to targeted antibacterial therapy, in pyogenic pericarditis can provide benefit to the patients and hasten their recovery [43].

There is considerable evidence that supports the use of colchicine in patients with pericarditis to improve remission rates and reduce recurrence rates when used adjunctly with NSAIDs compared to NSAIDs alone. Colchicine concentrates inside granulocytes whose proinflammatory molecules are inhibited by its actions. In addition, it blocks the tubulin polymerization and impairs microtubule assembly, further contributing to decreased inflammation [43, 44]. In a recent meta-analysis assessing different medical therapies for pericarditis, colchicine added to NSAIDs was associated with a reduced risk of treatment failure (OR, 0.23; 95% CI, 0.11–0.49) and recurrent pericarditis (OR, 0.39; 95% CI, 0.20–0.77) when compared to NSAIDs alone [45]. Another meta-analysis reported that the use of colchicine reduced risk of postpericardiotomy syndrome (OR, 0.48; 95% CI, 0.33–0.68) and recurrent pericarditis in patients with acute pericarditis (OR, 0.31; 95% CI, 0.19–0.52) [46].

In addition to antibiotics and anti-inflammatory drugs, the infusion of fibrinolytics has shown benefit in patients with fibropurulent pericarditis. Streptokinase or urokinase given intrapericardially can stop the drainage and decrease the risk of recurrence as well as complications. Adding corticosteroids to fibrinolytics has also been described as an alternative to surgical options [35, 41, 47, 48].

### 4.7. Surgical Management

Pericardial drainage is almost always indicated in patients who have massive pericardial effusion or in patients with small effusion not responsive to medical treatment. In addition, pericardial fluid drainage allows fluid cytology and analysis to be performed to identify the cause of the effusion. Other indications include the desire to administer a medication or drug into the pericardium [49]. In our patient, immediate pericardial drainage allowed 1.2 L of yellow fluid to be drained. However, the pericardial drain was not left in place for continuous drainage. After drainage, a repeat echocardiogram revealed only minimal residual pericardial effusion with normalization of vital signs and resolution of symptoms. As such, we were not prompted to keep a pericardial drain longer than medically necessary. In some cases, it may be medically indicated to keep a pericardial drain in the pericardium after evacuation of the fluid. If pericardiocentesis does not completely evacuate the fluid or if active secretion of fluid or bleeding is present and causes rapid reaccumulation of the effusion, then keeping a pericardial drain for a longer duration would be indicated. Continuous drainage of the pericardium is also necessary when more than 50 cc of fluid remains in the pericardium after fluid evacuation. Nonetheless, the catheter should be removed as soon as possible in order to minimize the risk of infection [50, 51]. Often, the above-mentioned approach does not properly eradicate the infection especially in cases where fibrin accumulation may lead to a pericardial empyema, constrictive pericarditis, persistent infection, or tamponade recurrence. In these cases, extensive pericardiectomy or pericardial window may be necessary. It is estimated that 40% of patients with bacterial pericarditis resort to surgical interventions for complete resolution [4, 52]. Immediate pericardiocentesis is life-saving but complete drainage is best achieved by surgical resection of the pericardium [20, 33]. Controversy is present regarding the best surgical approach. Several surgical options exist such as placement of a pericardial window with pleural drainage, subxiphoid tube drainage, partial pericardiectomy with pericardial drainage, and total pericardiectomy. Subxiphoid pericardiotomy allows the surgeon to manually remove adhesions while establishing a pericardial window [1, 20, 53]. Selection of the best technique is multifactorial and depends primarily on the cause of pericarditis, fluid viscosity, clinical improvement after medical management, and the experience of the surgeon. Combining both medical and surgical approaches decreases mortality well below 20% [4, 52]. We believe that early diagnosis and prompt medical therapy in our patient played an important role in the resolution of the pericarditis and his symptoms without resorting to a surgical intervention.

## 5. Conclusion

Bacterial pericarditis is a serious diagnosis that requires prompt diagnosis and treatment to avoid fatal complications. Pericarditis caused by *Cutibacterium acnes* is widely underreported. Isolation of this organism from pericardial fluid should not be disregarded as a skin contaminant especially in patients with a history of dental surgeries or implanted devices. The culture should be kept in the la for several days. Larger prospective studies are needed to examine the true incidence of *C. acnes* infection in patients presenting with pericardial effusion that have been otherwise misdiagnosed as idiopathic.

## Figures and Tables

**Table 1:** Summary characteristics of patients with pericarditis caused by *Cutibacterium acnes*.

Author (year)	Sex, age	Medical history	Diagnostic delay	Echocardiography results	Medical treatment, duration	Surgical treatment	Outcome
Iseki (1998) [9]	M, 62	Hepatitis B, tooth decay	3 months	Constrictive pericarditis	None	Total pericardiectomy	Resolved

Parikh (2009) [10]	F, 46	Hepatitis C, tooth decay, substance abuse	NA	Tamponade	Pen G-7 w	Pericardial window	Resolved

Mesado et al. (2013) [11]	M, 55	Respiratory tract infection	2 months	Constrictive pericarditis	Cef + AC: 1 m; Amox + steroids: 6 m	Subtotal pericardiectomy	1 relapse, resolved in 1 year
	M, 26	Dental caries	2 weeks	Tamponade	AC: 6 m; Dox: 2 m Steroids: 3 m	Pericardial window surgical drainage	1 relapse, resolved in 21m
	M, 31	Dental infection	9 months	Constrictive pericarditis	Pen G + Amox: 10 m; Mox: 8 m; Steroids + NSAIDs + colchicine:10 m	Subtotal pericardiectomy, patched epicardiectomy	Resolved at 3y
	M, 72	NA	20 months	Pericardial thickening	Cef: 2 w; Minocycline: 2 m	Total pericardiectomy	Resolved
	F, 38	Respiratory tract infection	1 month	Constrictive pericarditis	Cef + deptomycin: 2 w; Dox: 6 m	Subxiphoid pericardial drainage	2 relapses

Cruz et al. (2015) [12]	M, 61	Coronary artery disease	5 months	Constrictive pericarditis	Amox: 3 w; PenG + colchicine + Dox: 6 w	None	1 relapse, resolved in 10w

Jensen (2017)	M, 75	Hypertension, heart failure	2 months	Concentric pericarditis	Pen G: 4 w; AC + NSAIDs: 12 w	Subtotal pericardiectomy	Resolved

Farhat (2018) [2]	M, 71	NA	3 weeks	Tamponade	Vancomycin: 2 w	Subxiphoid pericardial drainage	Resolved

Arabi (2019)	M, 20	ASD	2 weeks	Tamponade	Pen G: 3 w Amox: 3 w	None	Resolved

Amox: amoxicillin; AC : amoxicillin-clavulanic acid; ASD : atrial septal defect; Cef: ceftriaxone; Dox: doxycycline; Mox: moxifloxacin; NA : not available; NSAIDs: nonsteroidal anti-inflammatory agents; Pen: penicillin.
